# Targeting coping strategies, emotion regulation, and self‐esteem in children with dyslexia to support mental health: A randomized‐controlled trial of *Clever Kids*

**DOI:** 10.1111/bjep.70122

**Published:** 2026-08-21

**Authors:** Mark Boyes, Kirsten Melville, Mandy Nayton, Hannah Pizzey, Suze Leitão

**Affiliations:** ^1^ Curtin enAble Institute Curtin University Perth Western Australia Australia; ^2^ School of Population Health Curtin University Perth Western Australia Australia; ^3^ The Dyslexia‐SPELD Foundation Perth Western Australia Australia; ^4^ School of Allied Health Curtin University Perth Western Australia Australia

**Keywords:** coping, dyslexia, emotion regulation, mental health, RCT, self‐esteem

## Abstract

**Background:**

Children with dyslexia are at elevated risk of mental health difficulties, both internalizing (emotional) and externalizing (behavioural) problems. *Clever Kids* is a nine‐week socio‐emotional well‐being programme developed specifically to support upper primary school children with dyslexia.

**Aims:**

We conducted a pre‐registered randomized‐controlled trial to test the efficacy of *Clever Kids*.

**Method:**

Seventy‐eight children (*M*_*age*_ = 10.19 years, 54% female) with clinically diagnosed dyslexia took part in the trial. Children were randomized to either attend *Clever Kids* (*n* = 38) or to a wait‐list control condition (*n* = 40). Coping skills, emotion regulation, self‐esteem, perseverance, assertiveness, as well as mental health (internalizing and externalizing symptoms) were measured at pre‐programme, post‐programme and at three‐month follow‐up using well‐validated measures.

**Results:**

The predicted interactions between intervention condition and time were significant for six outcomes. Attending *Clever Kids* was associated with significant improvements in child‐reported coping, emotion regulation, academic and social self‐esteem, as well as parent‐reported anxiety and depression. Effects were all maintained at three‐month follow‐up. Unexpectedly, being assigned to the wait‐list condition was associated with improvements in perseverance at follow‐up assessment. For all other outcomes, interactions between intervention condition and time were non‐significant.

**Conclusion:**

*Clever Kids* appears to be a promising programme for supporting socio‐emotional well‐being of children with dyslexia. Future research should consider incorporating caregivers more explicitly into the programme and alternative delivery modes, which could expand the reach of the programme.

## INTRODUCTION

Around 10% of children have significant difficulties learning to read (Snowling, 2008), representing 1–3 children in a typical Australian classroom. Along with reading difficulties, children with dyslexia are also at elevated risk of mental health difficulties, both internalizing (emotional) and externalizing (behavioural) problems (Carroll et al., 2005; Francis et al., 2019). Importantly, longitudinal studies demonstrate reading difficulties are prospectively associated with internalizing and externalizing symptoms and a risk factor for the development of mental health problems (Robidoux et al., 2024). While service‐providers have identified socio‐emotional well‐being as a significant area of unmet need, with increasing parent demand for services, there has been little attempt to develop and evaluate mental health interventions for children with dyslexia (Boyes et al., 2016; Francis et al., 2021). We tested the efficacy of a socio‐emotional well‐being programme developed specifically for children with dyslexia (*Clever Kids*; The Dyslexia‐SPELD Foundation – Literacy and Clinical Services, 2021).

A growing literature reports relationships between reading impairments and internalizing and externalizing difficulties, and recent meta‐analyses confirmed robust associations between poor reading and both anxiety and depression (Francis et al., 2019) as well as behavioural problems (Pickren et al., 2024). While evidence linking reading difficulties with worse mental health is accumulating, less research has investigated why this is the case (Boyes et al., 2016); however, several potential risk and protective factors have been identified. Specifically, mental health challenges among children with dyslexia have been linked to the use of less effective coping strategies (Alexander‐Passe, 2006; Leitão et al., 2017), difficulties regulating emotion (Boyes et al., 2020; Singer, 2005), lower self‐esteem (Boyes et al., 2020; Boyes et al., 2018; McArthur et al., 2020; Terras et al., 2009), as well as limited social support and difficulties with peer relationships (Boyes et al., 2020; Claessen et al., 2020; Leitão et al., 2017). Importantly, these factors are consistently associated with mental health in the broader child psychology literature and are well‐aligned with the Social–Emotional Learning Framework (Payton et al., 2000), which has been extensively used to underpin school‐based interventions supporting social–emotional well‐being. However, while whole‐school programmes have been shown to improve mental health outcomes (Neil & Christensen, 2009), their dependence on literacy skills to engage with the content may limit their effectiveness for children with reading difficulties in whole‐school settings.

*Clever Kids* is an adaptation of a whole‐school coping programme for upper primary school students (Firth et al., 2013). *Clever Kids* was developed by the Dyslexia‐SPELD Foundation (DSF)^1^ and implemented by DSF psychologists. The programme is delivered in small groups outside school hours, providing attendees opportunities to meet other children with dyslexia; this social aspect of *Clever Kids* was highlighted as a strength in a pilot evaluation of the programme (Boyes et al., 2021). *Clever Kids* targets children in their final two years of primary school as the transition to secondary school can be particularly stressful for children with dyslexia. The programme includes a combination of explicit instruction, modelling, role‐play and ongoing revision of concepts. Activities are designed to be accessible for children with dyslexia, and parents are informed of content so they can support children in practising skills.

The structure of *Clever Kids* is described in the programme manual (The Dyslexia‐SPELD Foundation – Literacy and Clinical Services, 2021). Briefly, *Clever Kids* consists of nine weekly sessions (~60 min) over a school‐term. The programme helps children understand and accept their dyslexia diagnosis and any associated difficulties. *Clever Kids* addresses a range of socio‐emotional constructs, including coping strategies (integrated into all programme sessions and a specific focus in Session 3), recognizing and regulating emotions (integrated into all programme sessions and a specific focus in Session 6), self‐esteem (particularly the idea that self‐esteem is multi‐dimensional and difficulties in one area may be independent of difficulties in other areas; Harter, 2012, Session 2), goal setting (Session 4), problem‐solving skills (Session 5) and perseverance (Session 5). The programme also addresses the role of assertiveness in developing and maintaining relationships, managing interpersonal conflict and asking for support when needed (Sessions 7 and 8). These constructs are all included or closely aligned with the key competencies of the Social–Emotional Learning Framework (Payton et al., 2000). *Clever Kids* has a particular emphasis on developing coping and emotion regulation skills, and these are a focus across all sessions.

A pilot study demonstrated the acceptability of *Clever Kids* to children with dyslexia and their families and highlighted the feasibility of conducting a larger trial (Boyes et al., 2021). This pilot also demonstrated programme attendance was associated with a significant reduction in the use of non‐productive coping strategies, such as avoidance and self‐blame. This was an important finding; a reliance on these types of strategies is associated with the development of mental health problems in children and adolescents (Guerra et al., 2016). There were also encouraging patterns regarding potential improvements in self‐esteem and internalizing symptoms; however, these effects were smaller, and the pilot was underpowered to detect them. Additionally, given logistical constraints, the pilot did not assess perseverance and assertiveness, key programme targets. The pilot was also limited to assessing global self‐esteem. Given evidence dyslexia is more strongly associated with specific facets of self‐esteem (e.g., academic self‐esteem; McArthur et al., 2020) and the multi‐dimensional approach *Clever Kids* takes regarding self‐esteem (Harter, 2012), it was suggested that future evaluations of the programme assess both global self‐esteem and specific dimensions of self‐esteem. Taken together, the findings of the pilot were encouraging and highlighted the need for a larger trial of the programme with a more comprehensive assessment of mechanisms targeted by the programme (Boyes et al., 2021).

The aim of this study was to test the efficacy of *Clever Kids* in improving socio‐emotional outcomes among children with dyslexia. Specifically, we predicted improvements in targeted mechanisms: coping, emotion regulation, self‐esteem (global, academic, social, behavioural), perseverance, and assertiveness. We also predicted reductions in emotional/internalizing and externalizing symptoms. Given the substantial focus on coping and emotion regulation skills across the programme, these were identified as the primary trial outcomes. All other outcomes were identified as secondary. The trial was pre‐registered (Australian New Zealand Clinical Trials Registry, ACTRN12623000371695).

## METHOD

### Design

We conducted a randomized‐controlled trial, powered to identify medium‐small intervention effects.^2^ Children were randomized to attend *Clever Kids* (intervention) or a wait‐list control condition. Participants were randomized at the individual level to condition and those allocated to the intervention attended one of four small intervention groups based on families' availability. Therefore, the design was partially nested, with clustering in the intervention arm (but not the control arm). Outcomes were assessed before randomization (pre‐programme), at programme completion (post‐programme) and three‐month follow‐up.

### Participants

Using the DSF database, we directly emailed parents of children with a dyslexia diagnosis who were in Year 5 or Year 6 and lived in the Perth metropolitan area (*n* ~ 590). Additionally, DSF tutors (*n* ~ 70) were asked to share the study with families. We recruited 80 families; however, two families withdrew from the study prior to any data collection (Figure 1). Seventy‐eight children (Male = 36; Female = 42, *M*_*age*_ = 10.19 years, *SD* = 0.66 years) and 78 caregivers (Mother = 71; Father = 5; Grandmother = 1) from a large Australian city participated in the study. Most children were of English, Irish, Scottish, Welsh (*n* = 43) or European (e.g., Italian, German, French; *n* = 34) ancestry. The sample included one Aboriginal child. Inclusion criteria included a diagnosis of dyslexia and children needed to be in the last two years of primary school (Year 5: *n* = 44; Year 6: *n* = 34).

**FIGURE 1:**
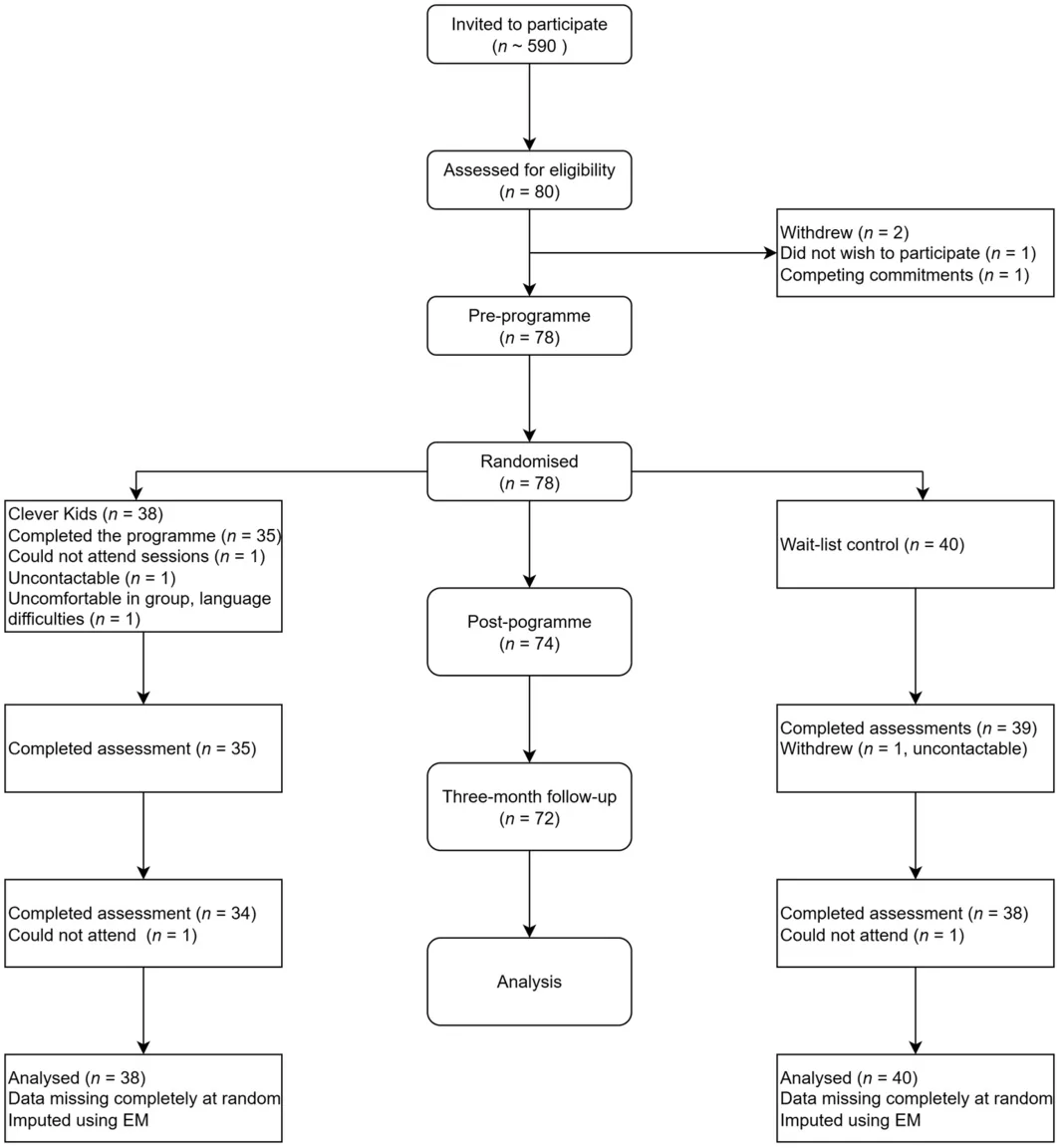
Participant flow diagram.

Dyslexia diagnoses had been established previously by DSF and were based on DSM‐5 criteria. Reading difficulties needed to have persisted for over six months despite provision of targeted and explicit intervention (American Psychiatric Association, 2013). After diagnosis, children are strongly encouraged to continue targeted literacy intervention. Children were not eligible if they were currently receiving psychological treatment or had additional complex needs (e.g., comorbid autism diagnosis, intellectual disability). Over the trial, four children dropped out of the study (Intervention: *n* = 3; Wait‐list: *n* = 1; Figure 1).

### Measures

*Primary outcomes* were assessed via child‐report. Coping skills were assessed with the short‐form of the Adolescent Coping Scale – 2nd Edition (Frydenberg & Lewis, 1993). The scale assesses the use of both productive and non‐productive coping strategies. Internal consistencies ranged between *α* = 0.64 (non‐productive coping, pre‐programme) and 0.81 (productive coping, three‐month follow‐up). We assessed emotion regulation difficulties with the Difficulties in Emotion Regulation Scale (Short‐form; Kaufman et al., 2016). This scale assesses difficulties in awareness and understanding of emotions, acceptance of emotions, ability to engage in goal‐directed behaviour (and refrain from impulsive behaviour) when experiencing negative emotions, and access to effective emotion regulation strategies. The total score provides an index of perceived difficulties in emotion regulation. Internal consistency in the current study ranged between *α* = 0.87 (pre‐programme, post‐programme) and *α* = 0.90 (three‐month follow‐up). Additionally, we assessed the use of two specific emotion regulation strategies (cognitive reappraisal, expressive suppression) with the Emotion Regulation Questionnaire for Children and Adolescents (Gullone & Taffe, 2012). Cognitive reappraisal refers to redefining an emotion‐eliciting situation so that its emotional impact is changed. Expressive suppression refers to inhibiting emotionally expressive behaviour. Cognitive reappraisal is associated with better mental health and expressive suppression is associated with poorer mental health (Hu et al., 2014). Internal consistencies ranged between *α* = 0.40 (expressive suppression, pre‐programme) and *α* = 0.85 (cognitive reappraisal, post‐programme).

*Secondary outcomes* were assessed via a combination of child‐ and parent‐report. We assessed global self‐esteem via child‐report with the Rosenberg Self‐Esteem Scale (Rosenberg, 1979). Internal consistency ranged between *α* = 0.83 (pre‐programme) and *α* = 0.88 (three‐month follow‐up). We assessed academic, social, and behavioural self‐esteem through child‐report with the scholastic competence, social competence and behavioural competence sub‐scales from the Self‐Perception Profile for Children (Harter, 2012). Internal consistencies ranged between *α* = 0.73 (scholastic competence, pre‐programme) and *α* = 0.85 (behavioural competence, three‐month follow‐up). We assessed perseverance via child‐report with the Grit Scale for Children and Adults (Sturman & Zappala‐Piemme, 2017). Internal consistency ranged between *α* = 0.74 (pre‐programme) and *α* = 0.84 (three‐month follow‐up). Child‐reported assertiveness was assessed with the assertion scale of the Social Skills Improvement System (Gresham & Elliott, 2008). Internal consistency ranged between α = 0.69 (pre‐programme) and α = 0.78 (three‐month follow‐up).

Mental health outcomes were assessed via both child‐ and parent‐report using the short‐form of the Revised Children's Anxiety and Depression Scale (RCADS‐25; Ebesutani et al., 2012) and the Strengths and Difficulties Questionnaire (SDQ; Goodman, 1997). The RCADS‐25 provides an index of both anxiety and depression. Internal consistencies for child‐reported RCADS‐25 ranged between *α* = 0.79 (depression, pre‐programme) and *α* = 0.88 (anxiety, pre‐programme). Internal consistencies for parent‐reported RCADS‐25 ranged between *α* = 0.69 (depression, post‐programme) and *α* = 0.82 (anxiety, pre‐programme). The SDQ (Goodman, 1997) contains five sub‐scales: emotional problems, conduct problems, hyperactivity, peer relationship problems and pro‐social behaviour. Internalizing symptoms scores are calculated by summing the emotional problems and peer problems sub‐scales. Externalizing symptoms scores are calculated by summing the conduct problems and hyperactivity sub‐scales. Internal consistencies for child‐reported internalizing and externalizing symptoms ranged between *α* = 0.69 (internalizing symptoms, pre‐programme) and *α* = 0.82 (externalizing symptoms, three‐month follow‐up). Internal consistencies for parent‐reported internalizing/externalizing symptoms ranged between *α* = 0.74 (internalizing symptoms, post‐programme) and *α* = 0.78 (internalizing symptoms, three‐month follow‐up).

### Procedure

Ethical approvals were obtained from the Curtin University Human Research Ethics Committee (HRE2023‐0034). Informed consent was obtained from caregivers, and informed assent was obtained for all child participants. The first author generated a computer‐based random number sequence to allocate 40 participant numbers to two conditions. No blocking was used and equal allocation was ensured by assigning the first half of the ordered sequence to one condition and the remainder to the other. The first author conducted all analyses and remained blinded to condition allocation until analyses were complete. The second author, who acted as programme facilitator and primary contact for families, enrolled participants into the trial and assigned them participant numbers sequentially based on order of enrolment. To determine which condition received the intervention, the second author conducted a coin toss to assign the one of the pre‐generated groups as the intervention condition. *Clever Kids* was delivered in four small groups (*n* = 8–11) by the second author (a DSF psychologist who manualized *Clever Kids* and developed and delivers the facilitator training). Additionally, two provisionally‐registered psychologists and one speech pathologist employed by DSF assisted with programme delivery. A detailed breakdown of programme attendance and homework completion is included in supplementary material (Tables S1 and S2). Attendance was generally good, with an average of 83% attendance at sessions (range: 68–87%). Homework completion was more variable, with an average of 67% completion for each activity (range: 32–97%). We administered child‐report measures in small groups (*n* ~ 4) with the help of research assistants who read items aloud. At all assessments, research assistants were blind to condition. Caregivers completed the SDQ and RCADS‐25 individually, although research assistants were available if needed. There was no cost to attend the programme (for both conditions), and children received a $15 gift voucher after each data collection session. Children in the wait‐list condition were offered the opportunity to attend *Clever Kids* after all data were collected (*n* = 35 attended). No adverse events occurred over the course of the trial.

## RESULTS

There was minimal missing data (~4.6% across the entire dataset) and Little's MCAR test indicated data were missing completely at random for all measures (all *p* ≥ .053). Therefore, missing data were imputed using expectation maximization. Child age and gender did not differ significantly between intervention and wait‐list conditions and were not adjusted for in the analyses. Additionally, the proportion of children who had previously or were currently receiving reading intervention did not differ significantly across conditions at any of the assessment points. At pre‐programme assessment, 89% of children had previously received reading intervention (Intervention: 90%; Wait‐list: 88%) and 64% were currently receiving reading intervention (Intervention: 68%; Wait‐list: 60%). Correlations between all study outcomes (overall and separately by condition) at pre‐programme, post‐programme, and three‐month follow‐up are included in supplementary materials (Tables S3–S8).

Efficacy of *Clever Kids* was tested in a series of generalized linear mixed models (GLMMs) in R4.4.2 using the lme4 package with restricted maximum likelihood estimation, which is preferred for small samples (Bates et al., 2015). Analyses were conducted at the individual level with participant included as a random effect. Condition (Intervention vs. Wait‐list), time (pre‐programme, post‐programme, three‐month follow‐up), and the condition*time interaction were included as fixed effects. We tested fixed effects using the lmerTest package (Kuznetsova et al., 2017) and used the r2GLMM package (Jaeger et al., 2016) to estimate effect sizes.^3^ Given multiple primary outcomes, *p* values for the condition*time interactions for primary outcomes were adjusted using the Holm procedure. Consistent with guidance that correction is required for primary outcomes, but secondary outcomes can be reported without adjustment (provided they are clearly identified and interpreted cautiously, e.g., FDA, 2022; Bender & Lange, 2001; Butcher et al., 2022), Holm adjustment was not applied to secondary outcomes. We calculated estimated marginal means for all outcomes (at each assessment point, disaggregated by condition—Table 1) using the emmeans package (Lenth, 2020). Finally, within each condition, planned comparisons (using a Holm adjustment for multiple comparisons) tested differences over time for all outcomes.^4^

**TABLE 1:** Estimated marginal means for primary and secondary outcomes over time (disaggregated by intervention condition).

	Clever Kids (*n* = 38)			Waitlist (*n* = 40)		
	Pre‐ *M* (SD)	Post‐ *M* (SD)	3‐month *M* (SD)	Pre‐ *M* (SD)	Post‐ *M* (SD)	3‐month *M* (SD)
	[95% CI]	[95% CI]	[95% CI]	[95% CI]	[95% CI]	[95% CI]
**Primary outcomes**						
Productive coping	33.84 (5.88) [31.76, 35.92]	33.39 (7.29) [31.32, 35.47]	33.16 (7.09) [31.07, 35.24]	34.65 (5.44) [32.62, 36.68]	33.73 (6.61) [31.70, 35.75]	33.85 (6.44) [31.82, 35.88]
Non‐productive coping	22.00 (4.79) [20.34, 23.66]	18.26 (5.68) [16.60, 19.92]	18.79 (5.14) [17.13, 20.45]	19.13 (4.72) [17.51. 20.74]	19.03 (5.48) [17.41, 20.64]	19.30 (5.18) [17.68, 20.92]
Difficulties in emotion regulation	48.37 (13.02) [44.36, 52.38]	43.18 (11.06) [39.17, 47.20]	43.45 (12.09) [39.43, 47.46]	39.93 (10.97) [36.01, 43.84]	42.80 (13.55) [38.89, 46.71]	40.95 (13.94) [37.04, 44.86]
Cognitive reappraisal	17.95 (3.84) [16.58, 19.31]	19.71 (5.63) [18.35, 21.07]	19.44 (4.76) [18.08, 20.81]	19.03 (3.81) [17.70, 20.35]	19.03 (3.50) [17.70, 20.35]	19.40 (3.65) [18.07, 20.73]
Expressive suppression	11.52 (2.77) [10.58, 12.47]	10.45 (3.83) [9.50, 11.39]	10.53 (3.67) [9.58, 11.47]	10.68 (2.03) [9.76, 11.59]	10.40 (2.63) [9.48, 11.32]	10.08 (2.35) [9.16, 10.99]
**Secondary outcomes**						
Global self‐esteem	17.84 (5.31) [16.05, 19.63]	19.84 (5.71) [18.05, 21.63]	20.45 (5.99) [18.67, 22.24]	18.80 (5.53) [17.05, 20.55]	19.55 (5.29) [17.80, 21.30]	20.48 (5.61) [18.73, 22.22]
Academic self‐esteem	12.61 (3.94) [11.29, 13.92]	13.94 (4.37) [12.63, 15.26]	14.47 (4.20) [13.16, 15.79]	13.52 (3.67) [12.24, 14.81]	14.48 (4.04) [13.19, 15.76]	13.55 (4.28) [12.27, 14.83]
Social self‐esteem	14.92 (3.73) [13.65, 16.19]	17.42 (4.04) [16.15, 18.69]	17.31 (3.96) [16.84, 19.31]	18.10 (4.04) [16.86, 19.34]	18.05 (4.00) [16.81, 19.29]	18.08 (3.95) [16.84, 19.31]
Behavioural self‐esteem	17.76 (3.66) [16.54, 18.99]	19.00 (3.38) [17.77, 20.23]	18.76 (3.78) [17.54, 19.99]	18.55 (3.95) [17.35, 19.75]	18.67 (3.62) [17.48, 19.87]	18.28 (4.45) [17.08, 19.47]
Perseverance	38.50 (6.11) [36.11, 40.89]	39.29 (8.14) [36.90, 41.68]	38.29 (8.08) [35.90, 40.68]	38.30 (7.10) [35.97, 40.63]	39.08 (7.70) [36.75, 41.40]	41.38 (7.29) [39.05, 43.70]
Assertiveness	18.87 (3.94) [17.73, 20.01]	19.08 (4.03) [17.94, 20.22]	19.47 (4.23) [18.33, 20.62]	19.48 (2.94) [18.36, 20.59]	18.48 (3.18) [17.36, 19.59]	19.80 (2.84) [16.69, 20.91]
Anxiety (child‐report)	13.71 (8.79) [11.30, 16.12]	12.05 (6.83) [9.64, 14.46]	11.05 (7.24) [8.64, 13.46]	10.63 (6.69) [8.28, 12.97]	10.48 (7.52) [8.13, 12.82]	9.98 (7.72) [7.63, 12.32]
Anxiety (parent‐report)	9.55 (5.54) [8.17, 10.93]	7.26 (3.91) [5.88, 8.65]	6.89 (3.96) [5.51, 8.28]	7.30 (4.00) [5.95, 8.65]	6.23 (3.30) [4.88, 7.57]	6.88 (4.75) [5.53, 8.22]
Depression (child‐report)	9.11 (4.94) [7.61, 10.60]	8.29 (4.30) [6.79, 9.79]	7.95 (4.08) [6.45, 9.45]	7.83 (4.67) [6.37, 9.38]	8.13 (4.83) [6.67, 9.58]	8.50 (5.05) [7.04, 9.96]
Depression (parent‐report)	7.45 (4.14) [6.33, 8.57]	5.34 (3.10) [4.22, 6.46]	5.89 (3.66) [4.78, 7.01]	6.00 (3.29) [4.91, 7.09]	5.45 (2.97) [4.36, 6.54]	5.90 (3.62) [4.81, 6.99]
Internalising (child‐report)	6.68 (3.18) [5.65, 7.72]	5.79 (3.38) [4.76, 6.82]	5.16 (2.84) [4.13, 6.19]	4.85 (3.29) [3.84, 5.86]	4.90 (3.46) [3.89, 5.91]	4.60 (3.07) [3.59, 5.61]
Internalising (parent‐report)	5.82 (3.49) [4.79, 6.84]	4.50 (3.38) [3.47, 5.23]	4.71 (3.76) [3.69, 5.74]	3.78 (2.94) [2.78, 4.77]	3.55 (2.64) [2.55, 4.55]	3.68 (2.82) [2.68, 4.67]
Externalising (child‐report)	7.82 (3.64) [6.64, 8.99]	6.97 (3.36) [5.80, 8.15]	7.58 (3.44) [6.40, 8.76]	7.18 (3.66) [6.03, 8.32]	7.15 (3.89) [6.00, 8.30]	6.88 (3.92) [5.73, 8.02]
Externalising (parent‐report)	7.71 (3.73) [6.64, 8.78]	6.89 (3.21) [5.83, 7.96]	7.29 (3.42) [6.22, 8.36]	7.70 (3.38) [6.66, 8.74]	7.10 (3.06) [6.06, 8.14]	7.70 (3.10) [6.66, 8.74]

As the design was partially nested, we conducted sensitivity analyses to test if clustering affected estimated effects. We ran partially nested mixed‐effects models for all outcomes, including a cluster‐level random intercept for intervention participants. Across both primary and secondary outcomes, the pattern of results was unchanged when clustering was accounted for. Therefore, we have retained the original participant‐level analyses in the paper as these align with the individual‐level randomization of the study.

### Primary outcomes

#### Coping

For productive coping, the effects of condition, time and the condition*time interaction were all non‐significant (Table 2). Within conditions, the effect of time was not significant in either the intervention [*F*(2, 74) = 0.27, *p* = 0.767, *f* ^2^ = 0.007] or wait‐list condition [*F*(2, 74) = 0.47, *p* = 0.627, *f* ^2^ = 0.012]. None of the planned comparisons were significant in either condition.

**TABLE 2:** Summary of inferential statistics and effect sizes from generalized linear mixed models for all primary and secondary outcomes.

	Condition				Time				Condition*time			
	*F*	*p*	*f* ^2^	*d*	*F*	*p*	*f* ^2^	*d*	*F*	*p*	*f* ^2^	*d*
Primary outcomes												
Productive coping	0.25	0.619	0.003	0.11	0.69	0.505	0.009	0.19	0.06	0.972	0.001	0.06
Non‐productive coping	0.32	0.573	0.004	0.13	5.59	**0.005**	0.074	0.54	5.63	**0.020**	0.074	0.54
Difficulties in emotion regulation	2.39	0.127	0.031	0.35	1.25	0.290	0.016	0.25	5.68	**0.020**	0.074	0.54
Cognitive reappraisal	0.02	0.889	0.000	0.00	2.96	0.055	0.038	0.39	2.13	0.366	0.028	0.34
Expressive suppression	0.69	0.410	0.009	0.19	3.33	**0.038**	0.044	0.42	0.72	0.970	0.009	0.19
Secondary outcomes												
Global self‐esteem	0.04	0.835	0.001	0.06	8.40	**<0.001**	0.111	0.67	.753	0.473	0.010	0.20
Academic self‐esteem	0.05	0.833	0.001	0.06	5.62	**0.004**	0.074	0.54	3.54	**0.032**	0.046	0.43
Social self‐esteem	3.66	0.060	0.048	0.44	7.60	**0.001**	0.100	0.63	8.10	**<0.001**	0.106	0.65
Behavioural self‐esteem	0.00	0.990	0.000	0.00	1.34	0.264	0.017	0.26	1.39	0.252	0.018	0.27
Perseverance	0.37	0.545	0.005	0.14	1.98	0.142	0.026	0.32	3.47	**0.034**	0.046	0.43
Assertiveness	0.03	0.888	0.000	0.00	3.46	**0.034**	0.046	0.43	1.87	0.157	0.025	0.32
Anxiety (child‐report)	1.57	0.215	0.020	0.28	3.37	**0.037**	0.044	0.42	1.34	0.264	0.017	0.26
Anxiety (parent‐report)	1.53	0.221	0.020	0.28	15.17	**<0.001**	0.200	0.89	5.44	**0.005**	0.072	0.54
Depression (child‐report)	0.10	0.749	0.001	0.06	0.21	0.809	0.003	0.11	2.17	0.117	0.029	0.34
Depression (parent‐report)	0.42	0.520	0.005	0.14	8.09	**<0.001**	0.110	0.66	3.40	**0.036**	0.045	0.42
Internalizing (child‐report)	2.95	0.090	0.038	0.39	4.25	**0.016**	0.060	0.49	2.36	0.098	0.031	0.35
Internalizing (parent‐report)	4.22	**0.043**	0.060	0.49	4.63	**0.011**	0.060	0.49	2.49	0.078	0.034	0.37
Externalizing (child‐report)	0.27	0.606	0.004	0.13	1.06	0.350	0.014	0.24	1.33	0.268	0.017	0.26
Externalizing (parent‐report)	0.09	0.765	0.001	0.06	3.12	**0.047**	0.041	0.40	0.26	0.770	0.003	0.11

*Note*: *p* values for primary outcomes have been adjusted using the Holm procedure. For all outcomes, significant *p* values are bolded.

For non‐productive coping, the effect of condition was not significant; however, the effect of time and the condition*time interaction were significant. Specifically, there was a significant effect of time in the intervention [*F*(2, 74) = 9.75, *p* < 0.001, *f* ^2^ = 0.264] but not the wait‐list [*F*(2, 78) = 0.06, *p* = 0.941, *f* ^2^ = 0.002] condition. For the intervention condition, there was a significant reduction in non‐productive coping between pre‐ and post‐programme assessment [*t*(152) = 4.31, *p* < .001, *d*_*RM*_ = 0.66] and this was maintained at three‐month follow‐up [*t*(152) = 3.71, *p* = .001, *d*_*RM*_ = 0.51]. None of the planned comparisons were significant in the wait‐list condition.

#### Emotion regulation

For perceived difficulties in emotion regulation, the effects of time and condition were non‐significant; however, the condition*time interaction was significant. Specifically, there was a significant effect of time in the intervention [*F*(2, 74) = 4.39, *p* = 0.016, *f* ^2^ = 0.119] but not in the wait‐list [*F*(2, 78) = 1.81, *p* = 0.170, *f* ^2^ = 0.046] condition. For the intervention condition, there was a significant reduction in perceived emotion regulation difficulties between pre‐ and post‐programme assessment [*t*(152) = 2.02, *p* = .012, *d*_*RM*_ = 0.37] and this was maintained at three‐month follow‐up [*t*(152) = 2.77, *p* = .013, *d*_*RM*_ = 0.32]. None of the planned comparisons were significant in the wait‐list condition.

Regarding specific emotion regulation strategies, for cognitive reappraisal the effects of condition, time and the condition*time interaction were all non‐significant. However, there was a significant effect of time within the intervention [*F*(2, 74) = 4.89, *p* = 0.010, *f* ^2^ = 0.132] but not the wait‐list [*F*(2, 78) = 0.25, *p* = 0.780, *f* ^2^ = 0.006] condition. For the intervention condition, there was a significant increase in cognitive reappraisal between pre‐ and post‐programme assessment [*t*(152) = 2.85, *p* = .015, *d*_*RM*_ = 0.61] and this was maintained at three‐month follow‐up [*t*(152) = 2.42, *p* = .033, *d*_*RM*_ = 0.53]. None of the planned comparisons were significant in the wait‐list condition. For expressive suppression, the effect of condition and the condition*time interaction were non‐significant. The overall effect of time was significant, but effects of time were not significant in either the intervention [*F*(2, 74) = 2.44, *p* = 0.094, *f* ^2^ = 0.066] or wait‐list [*F*(2, 78) = 1.16, *p* = 0.320, *f* ^2^ = 0.030] condition. None of the planned comparisons were significant in either condition.

### Secondary outcomes

#### Self‐esteem

For general self‐esteem, the effect of condition and the condition*time interaction were non‐significant. The overall effect of time was significant. There was a significant effect of time in the intervention [*F*(2, 74) = 5.58, *p* = 0.006, *f* ^2^ = 0.151] but not the wait‐list [*F*(2, 78) = 3.04, *p* = 0.053, *f* ^2^ = 0.078] condition. For the intervention condition, there was a significant increase in general self‐esteem between pre‐ and post‐programme assessment [*t*(152) = 2.64, *p* = .018, *d*_*RM*_ = 0.42] and this was maintained at three‐month follow‐up [*t*(152) = 3.44, *p* = .002, *d*_*RM*_ = 0.48]. None of the planned comparisons were significant in the wait‐list condition.

Regarding specific domains of self‐esteem, for academic self‐esteem the effect of condition was not significant; however, the effect of time and the condition*time interaction were significant. There was a significant effect of time within the intervention [*F*(2, 74) = 6.83, *p* = 0.002, *f* ^2^ = 0.185] but not the wait‐list [*F*(2, 78) = 2.24, *p* = 0.113, *f* ^2^ = 0.057] condition. For the intervention condition, there was a significant increase in academic self‐esteem between pre‐ and post‐programme assessment [*t*(152) = 2.57, *p* = .023, *d*_*RM*_ = 0.49] and this was maintained at three‐month follow‐up [*t*(152) = 3.57, *p* = .001, *d*_*RM*_ = 0.55]. None of the planned comparisons were significant in the wait‐list condition. For social self‐esteem, the effect of condition was not significant; however, the effect of time and the condition*time interaction were significant. There was a significant effect of time within the intervention [*F*(2, 74) = 13.11, *p* < 0.001, *f* ^2^ = 0.355] but not the wait‐list [*F*(2, 78) = 0.01, *p* = 0.994, *f* ^2^ = 0.057] condition. For the intervention condition, there was a significant increase in social self‐esteem between pre‐ and post‐programme assessment [*t*(152) = 4.89, *p* < .001, *d*_*RM*_ = 0.73] and this was maintained at three‐month follow‐up [*t*(152) = 4.69, *p* < .001, *d*_*RM*_ = 0.61]. None of the planned comparisons were significant in the wait‐list condition. For behavioural self‐esteem, the effects of condition, time and the condition*time interaction were all non‐significant. The effect of time was non‐significant in both the intervention [*F*(2, 74) = 2.18, *p* = 0.120, *f* ^2^ = 0.059] and wait‐list [*F*(2, 78) = 0.28, *p* = 0.758, *f* ^2^ = 0.007] conditions. None of the planned comparisons were significant in either condition.

#### Perseverance

The effects of condition and time were not significant; however, there was a significant condition*time interaction. Unexpectedly, the effect of time was significant in the wait‐list [*F*(2, 78) = 6.70, *p* = 0.002, *f* ^2^ = 0.172] but not intervention [*F*(2, 74) = 0.41, *p* = 0.663, *f* ^2^ = 0.111] condition. The wait‐list condition reported an increase in perseverance at three‐month follow‐up relative to both pre‐ [*t*(152) = 3.05, *p* = 0.008, *d*_*RM*_ = 0.495] and post‐programme assessments [*t*(152) = 2.28, *p* = 0.048, *d*_*RM*_ = 0.41]. None of the planned comparisons were significant in the intervention condition.

#### Assertiveness

The effect of condition and the condition*time interaction were non‐significant; however, there was a significant effect of time. There was a significant effect of time within the wait‐list [*F*(2, 78) = 5.04, *p* = 0.009, *f* ^2^ = 0.129] but not the intervention [*F*(2, 74) = 0.78, *p* = 0.461, *f* ^2^ = 0.021] condition. Follow‐up comparisons in the wait‐list condition indicated non‐significant changes in assertiveness between pre‐programme assessment and both post‐programme [*t*(152) = 2.19, *p* = 0.060, *d*_*RM*_ = 0.37] and three‐month follow‐up assessments [*t*(152) = 0.71, *p* = 0.78, *d*_*RM*_ = 0.13]. However, there was a significant increase between post‐programme and three‐month follow‐up assessments [*t*(152) = 2.90, *p* = 0.013, *d*_*RM*_ = 0.43]. None of the planned comparisons were significant in the intervention condition.

#### Anxiety

For child‐reported anxiety. The effect of condition and the condition*time interaction were non‐significant; however, there was a significant effect time. The effect of time was significant in intervention [*F*(2, 74) = 4.26, *p* = 0.018, *f* ^2^ = 0.115] but not the wait‐list [*F*(2, 78) = 0.30, *p* = 0.745, *f* ^2^ = 0.008] condition. In the intervention condition, there was a significant reduction in child‐reported anxiety scores from pre‐programme assessment to three‐month follow‐up [*t*(152) = 2.91, *p* = 0.013, *d*_*RM*_ = 0.41]. Differences between pre‐ and post‐programme assessment, as well as post‐programme assessment and three‐month follow‐up were non‐significant. None of the planned comparisons in the wait‐list condition were significant. For parent‐reported anxiety, the effect of condition was non‐significant; however, the effect of time and the condition*time interaction were significant. The effect of time was significant within both the intervention [*F*(2, 74) = 15.07, *p* < 0.001, *f* ^2^ = 0.406] and the wait‐list condition [*F*(2, 78) = 3.12, *p* = 0.050, *f* ^2^ = 0.080]; however, the effect was substantially stronger in the intervention condition. In the intervention condition, there was a significant reduction in parent‐reported anxiety scores from pre‐ to post‐programme [*t*(152) = 4.72, *p* < 0.001, *d*_*RM*_ = 0.61], which was maintained at three‐month follow‐up [*t*(152) = 5.48, *p* < 0.001, *d*_*RM*_ = 0.69]. None of the planned comparisons were significant in the wait‐list condition.

#### Depression

For child‐reported depression, the effects of time, condition and the condition*time interaction were all non‐significant. The effect of time was not significant in either the intervention [*F*(2, 74) = 1.26, *p* = 0.290, *f* ^2^ = 0.34] or the wait‐list [*F*(2, 78) = 0.96, *p* = 0.387, *f* ^2^ = 0.025] condition and none of the planned comparisons were significant in either condition. For parent‐reported depression, the effect of condition was non‐significant; however, the effect of time and the condition*time interaction were significant. The effect of time was significant within the intervention [*F*(2, 74) = 8.80, *p* < 0.001, *f* ^2^ = 0.238] but not the wait‐list [*F*(2, 78) = 0.96, *p* = 0.386, *f* ^2^ = 0.025] condition. In the intervention condition, there was a significant reduction in parent‐reported depression scores from pre‐ to post‐programme assessment [*t*(152) = 4.41, *p* = 0.001, *d*_*RM*_ = 0.63], which was maintained at three‐month follow‐up [*t*(152) = 3.25, *p* = 0.003, *d*_*RM*_ = 0.41]. None of the planned comparisons in the wait‐list condition were significant.

#### Internalizing symptoms

For child‐reported internalizing symptoms, the effect of condition and the condition*time interaction were non‐significant; however, there was a significant effect of time. The effect of time was significant within the intervention [*F*(2, 74) = 6.01, *p* = 0.004, *f* ^2^ = 0.163] but not the wait‐list [*F*(2, 78) = 0.29, *p* = 0.747, *f* ^2^ = 0.007] condition. In the intervention condition, there was a significant reduction in child‐reported internalizing symptoms from pre‐programme assessment to three‐month follow‐up [*t*(152) = 3.50, *p* = 0.002, *d*_*RM*_ = 0.58]. Differences between pre‐ and post‐programme, as well as post‐programme and three‐month follow‐up assessments, were non‐significant. None of the planned comparisons in the wait‐list condition were significant. For parent‐reported internalizing symptoms the effects of condition and time were significant. The condition*time interaction was non‐significant. The effect of time was significant within the intervention [*F*(2, 74) = 6.46, *p* = 0.002, *f* ^2^ = 0.189] but not the wait‐list [*F*(2, 78) = 0.20, *p* = 0.823, *f* ^2^ = 0.005] condition. In the intervention condition, there was a significant reduction in parent‐reported internalizing symptoms from pre‐ to post‐programme assessment [*t*(152) = 3.45, *p* = 0.002, *d*_*RM*_ = 0.54], which was maintained at three‐month follow‐up [*t*(152) = 2.90, *p* = 0.009, *d*_*RM*_ = 0.43]. None of the planned comparisons in the wait‐list condition were significant.

#### Externalizing symptoms

For child‐reported externalizing symptoms, the effects of condition, time, and the condition*time interaction were all non‐significant. The effect of time was not significant in either the intervention [*F*(2, 74) = 1.65, *p* = 0.199, *f* ^2^ = 0.045] or the wait‐list [*F*(2, 78) = 0.40, *p* = 0.672, *f* ^2^ = 0.010] condition. None of the planned comparisons were significant in either the intervention of the wait‐list condition. For parent‐reported externalizing symptoms, the effect of condition and the condition*time interaction were non‐significant. Although, there was a significant overall effect of time, the effect of time was not significant in either the intervention [*F*(2, 74) = 1.70, *p* = 0.190, *f* ^2^ = 0.046] or the wait‐list [*F*(2, 78) = 1.66, *p* = 0.197, *f* ^2^ = 0.043] condition. None of the planned comparisons were significant in either the intervention or the wait‐list condition.

## DISCUSSION

In a pre‐registered randomized‐controlled trial we tested the efficacy of *Clever Kids*, a socio‐emotional well‐being programme developed for children with dyslexia in upper primary school. Specifically, we tested if programme attendance was associated with improvements in targeted mechanisms, including coping skills, difficulties in emotion regulation and emotion regulation strategies (primary outcomes), as well as self‐esteem, perseverance, assertiveness and emotional/internalizing and externalizing symptoms (secondary outcomes).

### Primary outcomes

*Clever Kids* attendance was associated with a significant reduction in non‐productive coping, which was maintained at three‐month follow‐up. Specifically, children who attended the programme reported reduced use of unhelpful strategies such as not telling people about their problems, avoiding problems and self‐blame. This replicates a smaller pilot evaluation of *Clever Kids* (Boyes et al., 2021). These consistent findings are important, as a reliance on non‐productive coping strategies is associated with the development of mental health problems in children and adolescents (Compas et al., 2001; Guerra et al., 2016). Extending previous results, the current trial also demonstrated that attending *Clever Kids* was associated with significant reductions in perceived emotion regulation difficulties, which was maintained at three‐month follow‐up. Again, this is an important finding as emotion regulation is robustly linked with child and adolescent mental health difficulties generally (Schäfer et al., 2017), as well as in the context of reading difficulties specifically (Boyes et al., 2020; Wilmot et al., 2023).

There were no intervention effects for specific emotion regulation strategies; however, it should be noted that for cognitive reappraisal, while non‐significant, the condition by time interaction fell just below our minimum detectable effect size. Additionally, children in the intervention condition (but not the wait‐list) did report significant increases in cognitive reappraisal which were maintained at three‐month follow‐up. These findings are consistent with expectations for effects that are close to the threshold of detectability. Importantly, such effects may not consistently reach statistical significance, particularly under more conservative analytic approaches, but may still reflect meaningful patterns of change (Hackshaw & Kirkwood, 2011; Gelman & Stern, 2006). Given that cognitive reappraisal is consistently associated with better child mental health (Schäfer et al., 2017), further research with larger samples would be valuable to improve precision of estimation and determine whether this borderline effect represents a reliable, albeit smaller, impact of the programme. Future research could also test if, over time, potential increases in cognitive reappraisal may translate into improvements in perceived emotion regulation ability (Doménech et al., 2024).

### Secondary outcomes

Given the number of outcomes tested, findings for secondary outcomes should be interpreted with caution; however, improvements in academic and social self‐esteem were particularly pleasing to observe. Previous research indicates reading difficulties may be more closely linked to specific domains of self‐esteem (in particular academic self‐esteem) than global self‐esteem (McArthur et al., 2020) and *Clever Kids* has a particular focus on developing a multi‐dimensional understanding of self‐esteem. Additionally, while the intervention effect for global self‐esteem was not significant, the condition by time interaction was small (and below our minimum detectable effect size), and children in the intervention condition (but not the wait‐list) did report significant increases in global self‐esteem that were maintained at three‐month follow‐up. While these findings should be interpreted with appropriate caution, they are encouraging and warrant testing with larger samples. It would also be interesting to test if, over longer time periods, increases in specific domains of self‐esteem translate into improvements in global self‐esteem (von Soest et al., 2016).

There were significant intervention effects for anxiety and depression symptoms (as reported by parents but not children). These reductions in children's anxiety and depression scores were maintained at three‐month follow‐up. Our findings extend those of the pilot study (Boyes et al., 2021) and are the first to demonstrate that *Clever Kids* may improve child emotional symptoms. However, because parents knew if their child had attended the programme, we cannot rule out the possibility that parent‐reports reflect expectancy effects. Future evaluations of the programme could consider supplementing reports from the primary caregiver with reports from another trusted adult who remains blind to condition (e.g., another family member or teacher). While there were no other significant intervention effects for mental health outcomes, it should again be noted that condition by time interactions for child‐reported anxiety and internalizing symptoms (child‐ and parent‐reported) fell just short of our minimum detectable effect. For all these outcomes, there were significant improvement the intervention condition (but not the wait‐list condition) that were maintained over time. Again, while these findings should be considered cautiously, they are promising and if confirmed in larger samples have the potential to add to the growing evidence base for *Clever Kids* in supporting the socio‐emotional well‐being of children with dyslexia.

### Limitations and directions for future research

While the study had many strengths, including a robust pre‐registered design, there are several limitations (of both the study and the programme) that should be noted. Regarding the study, while we used well‐validated measures for all outcomes, some reliabilities within our sample did not meet established cut‐offs. In particular, the reliability for expressive suppression was very low at pre‐programme assessment (α = 0.40) and findings for this outcome should be treated with extreme caution. Why reliabilities for some measures did not meet thresholds is unclear, but it is at least plausible that this may reflect reading‐related difficulties in comprehension. For example, Rossiter et al. (2014) has argued that measures designed for typically‐developing children may pose difficulties when used with children with learning difficulties, particularly in terms of appropriateness and comprehensibility of language and concepts within items. We argue detailed psychometric evaluation of measures of socio‐emotional well‐being in the context of reading difficulties is needed; in particular, establishing measurement invariance of measures across children with and without reading difficulties should be an urgent priority (Campbell et al., 2026). Additionally, as a funded trial, programme attendance was free, and children were reimbursed for attending data collection sessions. Whether observed effects are maintained under real‐world conditions remains an open question. Finally, the sample was predominantly of European ancestry, and all children had received a clinical diagnosis of dyslexia. Additional research is required to determine if findings generalize to children from other cultural backgrounds, and/or children with undiagnosed reading difficulties.

Regarding the programme, no intervention effects were observed for productive coping, specific emotion regulation strategies, assertiveness or externalizing symptoms in either the current study or the pilot (Boyes et al., 2021). Given these are key targets of the programme future research should explore whether these need to be assessed with alternative measures, or whether programme content needs to be adjusted to ensure children are learning the targeted skills. Detailed qualitative co‐design work with consumers could address questions regarding both measurement and programme content (Dunstan et al., 2024; Hyett et al., 2020). Unexpectedly, children who did not attend *Clever Kids* reported improvements in perseverance; driven by a change between post‐programme and follow‐up assessments. We do not have any explanation for this effect; however, it may reflect variability in unmeasured external factors (e.g., participation in alternative school‐based social–emotional well‐being programmes or other non‐school‐related services/activities). Future evaluations should monitor perseverance in wait‐list conditions to see if this is an anomaly or a replicable effect and consider assessing a broader range of external factors that may account for this unexpected finding.

*Clever Kids* is a nine‐week programme with minimal parent involvement; including parents and identifying time‐efficient delivery modes could potentially improve outcomes and scalability (Leitão et al., 2025). For example, incorporating concurrent sessions for parents (while children are attending sessions) and/or developing weekend intensive versions of the programme (that parents are invited to attend), may increase parent involvement and scalability. Relatedly, a camp‐based adaptation (attended by child and parent) has been developed and received excellent feedback (Leitão et al., 2025); however, rigorous evaluation of this camp is still required. The programme could also be adapted for use with other priority age groups. As mental disorders typically onset across adolescence (Kessler et al., 2007), and transition from school into work can be particularly stressful for individuals with reading difficulties, we could collaborate with adolescents and young adults to adapt *Clever Kids* to meet their needs.

Finally, *Clever Kids* does not include literacy support. We believe the well‐being of children with dyslexia needs to be considered alongside high‐quality evidence‐based reading instruction (Boyes et al., 2021). While the proportion of children receiving reading intervention did not differ systematically across conditions, and most participants were receiving tutoring, it is important to note that around a third of children were not currently receiving tutoring. It is possible that a combination of both literacy and socio‐emotional intervention may be more effective, particularly for children not receiving targeted reading intervention. Current work is actively investigating the potential of combined approaches (Francis et al., 2021) and this is an important area for ongoing investigation.

## CONCLUSION

Service‐providers have identified socio‐emotional well‐being among children with dyslexia as a significant area of unmet need, with increasing parent demand for services. This study demonstrates the potential efficacy of *Clever Kids* in improving coping skills, emotion regulation, academic and social self‐esteem, as well as symptoms of anxiety and depression, among children with dyslexia in upper primary school. While more research is clearly needed, particularly in terms of real‐world implementation using larger samples, our findings contribute to an initial evidence base to underpin considerations around scalability and adaptations for other priority‐age groups.

## AUTHOR CONTRIBUTIONS

**Mark Boyes:** Conceptualization; methodology; formal analysis; data curation; funding acquisition; writing – review and editing; writing – original draft; project administration. **Kirsten Melville:** Methodology; writing – review and editing; conceptualization; project administration. **Mandy Nayton:** Conceptualization; methodology; writing – review and editing. **Hannah Pizzey:** Methodology; writing – review and editing; project administration. **Suze Leitão:** Conceptualization; writing – review and editing; methodology.

## CONFLICT OF INTEREST STATEMENT

Mandy Nayton is the CEO of the Dyslexia‐SPELD Foundation. Kirsten Melville is a registered psychologist employed at the Dyslexia‐SPELD Foundation. Suze Leitão is a Board Member of the Dyslexia‐SPELD Foundation. Mark Boyes is a member of the Dyslexia‐SPELD Foundation.

## Supporting information


**Table S1.** Intervention attendance by participant.
**Table S2.** Homework completion by participant.
**Table S3.** Correlations between all outcome variables at pretest (whole sample).
**Table S4.** Correlations between all outcome variables at pretest by condition.
**Table S5.** Correlations between all outcome variables at post‐test (whole sample).
**Table S6.** Correlations between all outcome variables at post‐test by condition.
**Table S7.** Correlations between all outcome variables at follow‐up (whole sample).
**Table S8.** Correlations between all outcome variables at follow‐up by condition.

## Data Availability

Data are available from the corresponding author upon reasonable request.

## Footnotes

^1^
DSF is the largest clinical service provider for children with learning difficulties in Western Australia, conducting over 1500 assessments annually.

^2^
Power analysis conducted using G*Power 3.1.9.4. Repeated measures design (within‐between interaction), two groups, three time‐points. Parameters: medium‐small effect size (*f* = 0.14; *f* ^2^ = 0.02), correlations among repeated measures (*r* = 0.55). Required *n* = 76 (38 per group) for 80% power (*α* = 0.05). However, our actual analyses used mixed models that included participant‐level random effects (and a Holm adjustment), which resulted in a slightly higher minimum detectable effect size for the condition*time interaction (*f* ^2^ = .04).

^3^
We report *f* ^2^ for effect size (Small: *f* ^2^ ~ 0.02, Medium: *f* ^2^ ~ 0.15, Large: *f* ^2^ ~ 0.35; Cohen, 1988).

^4^
We report *d*_*Repeated Measures*_ (*d*_*RM*_) for effect size (Small: *d*_*RM*_ ~ 0.20, Medium: *d*_*RM*_ ~ 0.50, Large: *d*_*RM*_ ~ 0.80). *d*_*RM*_ incorporates correlations between repeated assessments and uses standard deviation of the baseline assessment (as this value is not influenced by the intervention; Morris & DeShon, 2002).
