# Supplementary material for: Targeting coping strategies, emotion regulation, and self‐esteem in children with dyslexia to support mental health: A randomized‐controlled trial of Clever Kids
Source: Br J Educ Psychol. 2026 Aug 21;96(4):1828–45. doi: 10.1111/bjep.70122 (PMC13752631; doi:10.1111/bjep.70122)
Supplement: Supplementary file 1 [file BJEP-96-1828-s001.docx]

*Supplementary Table 1.* Intervention attendance by participant.

|  | | **Session** | | | | | | | | | | |  | |
| --- | --- | --- | --- | --- | --- | --- | --- | --- | --- | --- | --- | --- | --- | --- |
| **Participant** | | **1** | **2** | **3** | **4** | **5** | **6** | **7** | **8** | **9** | **Total Attended** | **%** | |  |
| 1 | 1 | | 1 | 1 | 1 | 1 | 1 | 1 | 1 | 1 | 9 | 100 | |  |
| 2 | 0 | | 1 | 1 | 1 | 1 | 0 | 1 | 0 | 1 | 6 | 66.7 | |  |
| 3 | 1 | | 1 | 1 | 0 | 1 | 1 | 2 | 2 | 2 | 8 | 88.9 | |  |
| 4 | 1 | | 1 | 1 | 1 | 1 | 1 | 1 | 1 | 1 | 9 | 100 | |  |
| 5 | 1 | | 1 | 1 | 1 | 1 | 1 | 1 | 1 | 1 | 9 | 100 | |  |
| 6 | 0 | | 0 | 1 | 1 | 1 | 1 | 0 | 0 | 1 | 5 | 55.6 | |  |
| 7 | 0 | | 0 | 1 | 1 | 0 | 1 | 0 | 1 | 1 | 5 | 55.6 | |  |
| 8 | 1 | | 1 | 1 | 1 | 1 | 1 | 1 | 0 | 0 | 7 | 77.8 | |  |
| 9 | 1 | | 1 | 1 | 1 | 1 | 1 | 1 | 0 | 1 | 8 | 88.9 | |  |
| 10 | 1 | | 1 | 1 | 1 | 1 | 1 | 1 | 1 | 1 | 9 | 100 | |  |
| 11 | 1 | | 0 | 0 | 0 | 0 | 0 | 0 | 0 | 0 | 1 | 11.2 | |  |
| 12 | 1 | | 1 | 1 | 1 | 1 | 1 | 1 | 1 | 1 | 9 | 100 | |  |
| 13 | 0 | | 0 | 1 | 1 | 1 | 1 | 1 | 1 | 1 | 7 | 77.8 | |  |
| 14 | 1 | | 1 | 1 | 1 | 1 | 1 | 1 | 0 | 0 | 7 | 77.8 | |  |
| 15 | 1 | | 1 | 0 | 1 | 1 | 1 | 1 | 0 | 1 | 7 | 77.8 | |  |
| 16 | 1 | | 1 | 1 | 1 | 1 | 1 | 1 | 1 | 1 | 9 | 100 | |  |
| 17 | 1 | | 1 | 1 | 1 | 1 | 1 | 1 | 1 | 1 | 9 | 100 | |  |
| 18 | 1 | | 1 | 1 | 1 | 1 | 1 | 0 | 1 | 1 | 8 | 88.9 | |  |
| 19 | 1 | | 1 | 0 | 1 | 1 | 1 | 1 | 1 | 1 | 8 | 88.9 | |  |
| 20 | 1 | | 0 | 1 | 1 | 1 | 1 | 1 | 1 | 1 | 8 | 88.9 | |  |
| 21 | 0 | | 1 | 1 | 1 | 0 | 0 | 0 | 0 | 0 | 3 | 33.4 | |  |
| 22 | 1 | | 0 | 1 | 1 | 0 | 1 | 1 | 1 | 0 | 6 | 66.7 | |  |
| 23 | 1 | | 1 | 1 | 0 | 1 | 1 | 1 | 1 | 1 | 8 | 88.9 | |  |
| 24 | 1 | | 1 | 1 | 1 | 1 | 0 | 1 | 1 | 1 | 8 | 88.9 | |  |
| 25 | 1 | | 1 | 1 | 1 | 1 | 1 | 1 | 0 | 1 | 8 | 88.9 | |  |
| 26 | 1 | | 1 | 1 | 1 | 1 | 0 | 1 | 0 | 1 | 7 | 77.8 | |  |
| 27 | 1 | | 1 | 1 | 1 | 0 | 1 | 0 | 0 | 0 | 5 | 55.6 | |  |
| 28 | 1 | | 1 | 1 | 1 | 1 | 1 | 1 | 1 | 1 | 9 | 100 | |  |
| 29 | 1 | | 1 | 1 | 1 | 1 | 1 | 1 | 1 | 0 | 8 | 88.9 | |  |
| 30 | 1 | | 1 | 1 | 1 | 1 | 1 | 1 | 1 | 1 | 9 | 100 | |  |
| 31 | 1 | | 1 | 1 | 1 | 1 | 1 | 1 | 1 | 1 | 9 | 100 | |  |
| 32 | 1 | | 1 | 1 | 1 | 1 | 1 | 1 | 1 | 1 | 9 | 100 | |  |
| 33 | 1 | | 1 | 1 | 1 | 1 | 1 | 1 | 1 | 1 | 9 | 100 | |  |
| 34 | 1 | | 1 | 1 | 0 | 1 | 1 | 1 | 1 | 1 | 8 | 88.9 | |  |
| 35 | 1 | | 1 | 0 | 1 | 1 | 1 | 1 | 1 | 1 | 8 | 88.9 | |  |
| 36 | 1 | | 1 | 0 | 1 | 1 | 0 | 1 | 0 | 1 | 6 | 66.7 | |  |
| 37 | 1 | | 1 | 1 | 1 | 1 | 1 | 1 | 1 | 1 | 9 | 100 | |  |
| 38 | 1 | | 0 | 1 | 1 | 1 | 1 | 1 | 1 | 1 | 8 | 88.9 | |  |
| Overall Session Attendance %/*M* | 86.8 | | 81.6 | 86.8 | 89.5 | 86.8 | 81.6 | 84.2 | 68.4 | 81.6 | 7.5 | 83.35 | |  |

Note: Values of 1 indicate attendance and values of 0 indicate non-attendance.

*Supplementary Table 2.* Homework completion by participant.

| **Session** | | | | | | | | | | | |
| --- | --- | --- | --- | --- | --- | --- | --- | --- | --- | --- | --- |
| **Participant** | | **2** | **3** | **4** | **5** | **6** | **7** | **8** | **Total Completed** | **%** |  |
| 1 | 1 | | 1 | 1 | 1 | 1 | 1 | 1 | 7 | 100 |  |
| 2 | 0 | | 1 | 1 | 1 | 0 | 0 | 0 | 3 | 42.9 |  |
| 3 | 1 | | 1 | 0 | 0 | 1 | 1 | 1 | 5 | 71.2 |  |
| 4 | 1 | | 1 | 1 | 1 | 1 | 0 | 1 | 6 | 85.7 |  |
| 5 | 1 | | 1 | 1 | 1 | 1 | 1 | 1 | 7 | 100 |  |
| 6 | 0 | | 0 | 1 | 1 | 1 | 0 | 0 | 3 | 42.9 |  |
| 7 | 0 | | 0 | 1 | 0 | 1 | 0 | 1 | 3 | 42.9 |  |
| 8 | 1 | | 1 | 1 | 1 | 1 | 0 | 0 | 5 | 71.2 |  |
| 9 | 1 | | 1 | 1 | 1 | 1 | 1 | 0 | 6 | 85.7 |  |
| 10 | 1 | | 1 | 1 | 1 | 1 | 0 | 1 | 6 | 85.7 |  |
| 11 | 0 | | 0 | 0 | 0 | 0 | 0 | 1 | 1 | 14.3 |  |
| 12 | 1 | | 1 | 0 | 1 | 1 | 0 | 1 | 6 | 85.7 |  |
| 13 | 0 | | 0 | 0 | 1 | 1 | 1 | 1 | 4 | 57.1 |  |
| 14 | 0 | | 1 | 1 | 1 | 1 | 0 | 0 | 4 | 57.1 |  |
| 15 | 0 | | 0 | 0 | 1 | 1 | 0 | 0 | 2 | 28.6 |  |
| 16 | 1 | | 1 | 0 | 1 | 1 | 0 | 0 | 4 | 57.1 |  |
| 17 | 1 | | 1 | 1 | 1 | 1 | 1 | 0 | 6 | 85.7 |  |
| 18 | 0 | | 1 | 0 | 1 | 1 | 0 | 0 | 4 | 57.1 |  |
| 19 | 0 | | 0 | 0 | 0 | 0 | 0 | 0 | 0 | 0 |  |
| 20 | 0 | | 0 | 0 | 1 | 1 | 1 | 0 | 3 | 42.9 |  |
| 21 | 1 | | 1 | 0 | 0 | 0 | 0 | 0 | 2 | 28.6 |  |
| 22 | 0 | | 0 | 0 | 1 | 0 | 0 | 0 | 1 | 14.3 |  |
| 23 | 0 | | 0 | 0 | 1 | 0 | 1 | 0 | 2 | 28.6 |  |
| 24 | 0 | | 1 | 0 | 1 | 0 | 0 | 0 | 2 | 28.6 |  |
| 25 | 1 | | 1 | 0 | 1 | 1 | 1 | 0 | 5 | 71.2 |  |
| 26 | 0 | | 1 | 0 | 1 | 0 | 0 | 0 | 2 | 28.6 |  |
| 27 | 1 | | 1 | 0 | 0 | 1 | 0 | 0 | 3 | 42.9 |  |
| 28 | 1 | | 1 | 0 | 1 | 1 | 1 | 0 | 5 | 71.2 |  |
| 29 | 1 | | 1 | 0 | 1 | 1 | 0 | 0 | 4 | 57.1 |  |
| 30 | 1 | | 1 | 0 | 1 | 1 | 1 | 0 | 5 | 71.2 |  |
| 31 | 1 | | 1 | 0 | 1 | 1 | 1 | 0 | 5 | 71.2 |  |
| 32 | 1 | | 1 | 0 | 1 | 1 | 1 | 0 | 5 | 71.2 |  |
| 33 | 1 | | 1 | 0 | 1 | 1 | 1 | 0 | 5 | 71.2 |  |
| 34 | 1 | | 1 | 0 | 1 | 1 | 1 | 0 | 5 | 71.2 |  |
| 35 | 1 | | 0 | 0 | 1 | 1 | 0 | 0 | 4 | 57.1 |  |
| 36 | 1 | | 0 | 0 | 1 | 0 | 0 | 0 | 2 | 28.6 |  |
| 37 | 1 | | 1 | 0 | 1 | 1 | 0 | 0 | 4 | 57.1 |  |
| 38 | 0 | | 0 | 0 | 1 | 1 | 1 | 0 | 3 | 42.9 |  |
| Overall Homework Completion %/*M* | 60.5 | | 68.4 | 47.4 | 84.2 | 76.3 | 42.1 | 31.6 | 3.9 | 56.0 |  |

Note: Values of 1 indicate homework completion, and values of 0 indicate homework not completed. Homework activities were assigned from Session 1 and returned the following session.

*Supplementary Table 3.* Correlations between all outcome variables at pretest (whole sample)

|  | **1** | **2** | **3** | **4** | **5** | **6** | **7** | **8** | **9** | **10** | **11** | **12** | **13** | **14** | **15** | **16** | **17** | **18** |
| --- | --- | --- | --- | --- | --- | --- | --- | --- | --- | --- | --- | --- | --- | --- | --- | --- | --- | --- |
| **Primary outcomes** | | | | | | | | | | | | | | | | | | |
| Productive coping | — |  |  |  |  |  |  |  |  |  |  |  |  |  |  |  |  |  |
| Non-productive coping | -.006 | — |  |  |  |  |  |  |  |  |  |  |  |  |  |  |  |  |
| Difficulties in emotion regulation | **-.252*** | **.726***** | — |  |  |  |  |  |  |  |  |  |  |  |  |  |  |  |
| Cognitive reappraisal | .190 | -.075 | **-.253*** | — |  |  |  |  |  |  |  |  |  |  |  |  |  |  |
| Expressive suppression | **-.241*** | .173 | .196 | **-.324**** | — |  |  |  |  |  |  |  |  |  |  |  |  |  |
| **Secondary outcomes** | | | | | | | | | | | | | | | | | | |
| Global self- esteem | **.426***** | **-.349**** | **-.514***** | .059 | -.201 | — |  |  |  |  |  |  |  |  |  |  |  |  |
| Academic self- esteem | .119 | **-.418***** | **-.403***** | -.028 | .131 | **.448***** | — |  |  |  |  |  |  |  |  |  |  |  |
| Social self- esteem | **.348**** | **-.340**** | **-.465***** | .**302**** | -.136 | **.302**** | **.278*** | — |  |  |  |  |  |  |  |  |  |  |
| Behavioural self-esteem | **.332**** | **-.286*** | **-.516***** | **.225*** | .205 | **.284*** | **.275*** | **.461***** | — |  |  |  |  |  |  |  |  |  |
| Perseverance | **.446***** | **-.369***** | **-.369***** | **.231*** | -.102 | **.453***** | **.445***** | **.354**** | **.479***** | — |  |  |  |  |  |  |  |  |
| Assertiveness | **.489***** | .220 | .053 | .220 | **-.242*** | .127 | -.115 | .125 | .108 | **.268*** | — |  |  |  |  |  |  |  |
| Anxiety (child-report) | -.116 | **.578***** | **.623***** | -.034 | -.015 | **-.493***** | **-.516***** | **-.451***** | **-.433***** | **-.373***** | .198 | — |  |  |  |  |  |  |
| Anxiety (parent-report) | .019 | .220 | **.252*** | .143 | -.147 | -.153 | -.207 | **-.305**** | -.211 | -.140 | .107 | **.413***** | — |  |  |  |  |  |
| Depression (child-report) | -.180 | **.537***** | **.649***** | **-.265*** | -.002 | **-.448***** | **-.561***** | **-.574***** | **-.550***** | **-.474***** | .092 | **.762***** | .196 | — |  |  |  |  |
| Depression (parent-report) | -.063 | .196 | **.290*** | -.046 | -.045 | -.139 | -.184 | **-.463***** | **-.385***** | **-.264*** | .011 | **.372***** | **.613***** | **.471***** | — |  |  |  |
| Internalising (child-report) | -.205 | **.528***** | **.558***** | -.121 | .272* | **-.491***** | **-.396***** | **-.527***** | **-.320**** | **-.326**** | .081 | **.708***** | **.334**** | **.597***** | **.280*** | — |  |  |
| Internalising (parent-report) | -.010 | **.342**** | **.338**** | -.044 | .128 | -.212 | -.157 | **-.490***** | **-.254*** | -.151 | .112 | **.416***** | **.694***** | **.349**** | **.697***** | **.376***** | — |  |
| Externalising (child-report) | -.214 | **.372***** | **.518***** | -.180 | -.083 | **-.344**** | **-.497***** | **-.363**** | **-.570***** | **-.624***** | .028 | .**509***** | .141 | .**616***** | **.310**** | **.417***** | .135 | — |
| Externalising (parent-report) | -.020 | **.281*** | **.381***** | -.019 | -.059 | -.059 | .013 | **-.296**** | **-.442***** | **-.264*** | .037 | **.225*** | **.417***** | **.316**** | **.624***** | .129 | **.426***** | **.290**** |

*Note. N* = 78. *p* < .05 = *, *p* < .01 = **, *p* < .001 = ***; significant correlations are bolded for clarity.

*Supplementary Table 4*. Correlations between all outcome variables at pretest by condition

|  | **1** | **2** | **3** | **4** | **5** | **6** | **7** | **8** | **9** | **10** | **11** | **12** | **13** | **14** | **15** | **16** | **17** | **18** | **19** |
| --- | --- | --- | --- | --- | --- | --- | --- | --- | --- | --- | --- | --- | --- | --- | --- | --- | --- | --- | --- |
| **Primary outcomes** | | | | | | | | | | | | | | | | | | | |
| Productive coping | — | -.077 | -.260 | .104 | -.252 | .255 | .144 | **.521***** | **.438**** | **.507***** | **.422**** | -.180 | .098 | **-.356*** | -.231 | -.197 | -.148 | -.287 | -.187 |
| Non-productive coping | .105 | — | **.698***** | -.220 | .298 | **-.356*** | **-.444**** | **-.397*** | **-.449**** | **-.406**** | .034 | **.458**** | .058 | **.565***** | .120 | **.529***** | .270 | **.531***** | **.316*** |
| Difficulties in emotion regulation | -.228 | **.700***** | — | **-.386*** | **.429**** | **-.573***** | **-.336*** | **-.627***** | **-.707***** | **-.410**** | -.033 | **.563***** | .203 | **.663***** | .329* | **.611***** | **.383*** | **.602***** | .558*** |
| Cognitive reappraisal | .257 | .154 | -.076 | — | **-.337*** | .249 | .069 | **.352*** | **.383*** | .271 | -.054 | -.243 | .170 | **-.524***** | -.231 | -.227 | -.231 | -.295 | -.245 |
| Expressive suppression | -.221 | .002 | -.036 | -.290 | — | **-.332*** | .030 | **-.481**** | -.163 | -.226 | -.158 | .229 | -.187 | **.437**** | .200 | **.442**** | **.318*** | .156 | .300 |
| **Secondary outcomes** | | | | | | | | | | | | | | | | | | | |
| Global self- esteem | **.595***** | **-.321*** | **-.469**** | -.173 | -.079 | — | **.483**** | **.519***** | **.357*** | **.406**** | .087 | **-.617***** | -.232 | **-.565***** | -.159 | **-.624***** | **-.391*** | **-.323*** | -.173 |
| Academic self- esteem | .081 | **-.364*** | **-.431**** | -.157 | .248 | **.402*** | — | .275 | **.314*** | **.573***** | -.024 | **-.389*** | -.123 | **-.417**** | -.089 | **-.408**** | -.105 | **-.576***** | -.049 |
| Social self- esteem | .168 | -.103 | -.163 | .180 | .260 | .023 | .229 | — | **.597***** | **.416**** | .264 | **-.506***** | -.113 | **-.688***** | **-.433**** | **-.511***** | **-.594***** | -.293 | -.466** |
| Behavioural self-esteem | .212 | -.066 | **-.335*** | .022 | **.563***** | .182 | .217 | .287 | — | **.543***** | .158 | **-.559***** | -.098 | **-.669***** | **-.421**** | **-.428**** | -.303 | **-.598***** | -.478** |
| Perseverance | **.387*** | **-.374*** | **-.393*** | .193 | -.003 | **.521***** | .315 | **.354*** | **.405*** | — | **.340*** | **-.439**** | -.005 | **-.495**** | -.172 | -.286 | -.156 | **-.745***** | -.297 |
| Assertiveness | **.537***** | **.435**** | .169 | **.419**** | -.274 | .151 | -.204 | -.037 | .054 | .221 | — | -.034 | -.074 | -.095 | -.252 | .047 | -.017 | .059 | -.241 |
| Anxiety (child-report) | -.048 | **.640***** | **.631***** | .182 | -.216 | **-.388*** | **-.598***** | **-.348*** | -.318 | **-.349*** | **.372*** | — | **.348*** | **.694***** | .292 | **.622***** | **.488**** | **.460**** | .184 |
| Anxiety (parent-report) | -.006 | .246 | .184 | .196 | -.201 | -.065 | -.233 | **-.356*** | -.279 | -.282 | .247 | **.406*** | — | .076 | **.407**** | .111 | **.503***** | .019 | .305 |
| Depression (child-report) | -.003 | **.486**** | **.635***** | .020 | **-.366*** | -.316 | **-.682***** | **-.447**** | **-.413**** | **-.468**** | .255 | **.812***** | .243 | — | **.550***** | **.576***** | **.572***** | **.506***** | **.453**** |
| Depression (parent-report) | .089 | .174 | .183 | .156 | -.254 | -.097 | -.224 | **-.436**** | **-.341*** | **-.379*** | .204 | **.383*** | **.709***** | **.388*** | — | .192 | **.575***** | .188 | **.702***** |
| Internalising (child-report) | -.189 | **.442**** | **.431**** | .065 | .078 | **-.333*** | **-.354*** | **-.432**** | -.160 | **-.417**** | .163 | **.767***** | **.438**** | **.602***** | .286 | — | **.434**** | **.401*** | .165 |
| Internalising (parent-report) | .145 | .286 | .173 | .201 | -.076 | -.016 | -.145 | -.268 | -.173 | -.174 | .255 | .309 | **.785***** | .122 | **.760***** | .216 | — | .147 | **.383*** |
| Externalising (child-report) | -.133 | .189 | **.452**** | -.039 | -.301 | **-.357*** | **-.409*** | **-.432**** | **-.529***** | **-.487**** | .019 | **.547***** | .208 | **.718***** | **.398*** | **.420**** | .084 | — | **.330*** |
| Externalising (parent-report) | .128 | .273 | .280 | .195 | -.312 | .054 | .069 | -.172 | **-.413**** | -.233 | .236 | .263 | **.518***** | .198 | **.589***** | .104 | **.499**** | .255 | — |

*Note.* Correlations above the diagonal are for the control group (*n* = 40); correlations below the diagonal are for the intervention group (*n* = 38). *p* < .05 = *, *p* < .01 = **, *p* < .001 = ***; significant correlations are bolded for clarity.

*Supplementary Table 5.* Correlations between all outcome variables at post-test (whole sample)

|  | **1** | **2** | **3** | **4** | **5** | **6** | **7** | **8** | **9** | **10** | **11** | **12** | **13** | **14** | **15** | **16** | **17** | **18** |
| --- | --- | --- | --- | --- | --- | --- | --- | --- | --- | --- | --- | --- | --- | --- | --- | --- | --- | --- |
| **Primary outcomes** | | | | | | | | | | | | | | | | | | |
| Productive coping | — |  |  |  |  |  |  |  |  |  |  |  |  |  |  |  |  |  |
| Non-productive coping | **-.346**** | — |  |  |  |  |  |  |  |  |  |  |  |  |  |  |  |  |
| Difficulties in emotion regulation | **-.385***** | **.667***** | — |  |  |  |  |  |  |  |  |  |  |  |  |  |  |  |
| Cognitive reappraisal | **.440***** | -.146 | -.216 | — |  |  |  |  |  |  |  |  |  |  |  |  |  |  |
| Expressive suppression | **-.224*** | **.408***** | **.481***** | **-.374***** | — |  |  |  |  |  |  |  |  |  |  |  |  |  |
| **Secondary outcomes** | | | | | | | | | | | | | | | | | | |
| Global self- esteem | **.564***** | **-.614***** | **-.594***** | **.247*** | **-.343**** | — |  |  |  |  |  |  |  |  |  |  |  |  |
| Academic self- esteem | **.355**** | **-.406***** | **-.456***** | .061 | -.099 | **.627***** | — |  |  |  |  |  |  |  |  |  |  |  |
| Social self- esteem | **.227*** | **-.531***** | **-.559***** | .210 | **-.412***** | **.449***** | **.355**** | — |  |  |  |  |  |  |  |  |  |  |
| Behavioural self-esteem | **.385***** | **-.403***** | **-.560***** | .137 | **-.269*** | **.345**** | **.281*** | .089 | — |  |  |  |  |  |  |  |  |  |
| Perseverance | **.530***** | **-.487***** | **-.635***** | **.305**** | **-.490***** | **.575***** | **.419***** | **.523***** | **.369***** | — |  |  |  |  |  |  |  |  |
| Assertiveness | **.555***** | **-.237*** | **-.278*** | **.429***** | **-.265*** | **.417***** | .113 | **.231*** | **.278*** | **.453***** | — |  |  |  |  |  |  |  |
| Anxiety (child-report) | -.149 | **.552***** | **.647***** | .062 | **.281*** | **-.493***** | **-.512***** | **-.448***** | **-.247*** | **-.329**** | .095 | — |  |  |  |  |  |  |
| Anxiety (parent-report) | -.055 | **.314**** | .148 | .133 | .049 | -.182 | -.107 | **-.266*** | .021 | -.193 | .012 | **.314**** | — |  |  |  |  |  |
| Depression (child-report) | **-.278*** | **.580***** | **.625***** | -.222 | **.315**** | **-.468***** | **-.445***** | **-.521***** | **-.425***** | **-.523***** | -.030 | **.671***** | **.230*** | — |  |  |  |  |
| Depression (parent-report) | -.162 | **.258*** | .196 | -.080 | **.229*** | -.168 | -.123 | **-.257*** | -.164 | **-.326**** | .024 | .150 | **.618***** | **.395***** | — |  |  |  |
| Internalising (child-report) | **-.303**** | **.623***** | **.654***** | -.119 | **.270*** | **-.586***** | **-.581***** | **-.539***** | **-.263*** | **-.578***** | -.108 | **.764***** | **.333**** | **.662***** | **.244*** | — |  |  |
| Internalising (parent-report) | .001 | .222 | .202 | -.153 | .177 | -.169 | -.080 | **-.392***** | .153 | **-.254*** | -.046 | **.260*** | **.606***** | **.312**** | **.603***** | **.415***** | — |  |
| Externalising (child-report) | **-.268*** | **.453***** | **.612***** | -.037 | **.244*** | **-.416***** | **-.516***** | **-.314**** | **-.596***** | **-.557***** | -.053 | **.561***** | .197 | **.606***** | **.303**** | **.611***** | .131 | — |
| Externalising (parent-report) | -.181 | **.267*** | **.289*** | -.148 | .098 | -.133 | -.083 | **-.226*** | **-.406***** | -.196 | -.106 | .157 | **.297**** | **.326**** | **.468***** | .192 | **.336**** | **.427***** |

*Note. N* = 78. *p* < .05 = *, *p* < .01 = **, *p* < .001 = ***; significant correlations are bolded for clarity.

*Supplementary Table 6.* Correlations between all outcome variables at post-test by condition

|  | **1** | **2** | **3** | **4** | **5** | **6** | **7** | **8** | **9** | **10** | **11** | **12** | **13** | **14** | **15** | **16** | **17** | **18** | **19** |
| --- | --- | --- | --- | --- | --- | --- | --- | --- | --- | --- | --- | --- | --- | --- | --- | --- | --- | --- | --- |
| **Primary outcomes** | | | | | | | | | | | | | | | | | | | |
| Productive coping | — | -.188 | **-.489**** | **.624***** | **-.334*** | **.562***** | .237 | **.411**** | **.395*** | **.464**** | **.459**** | -.281 | .226 | **-.315*** | .064 | -.284 | .037 | **-.415**** | -.128 |
| Non-productive coping | -.291 | — | **.629***** | -.273 | .273 | **-.490**** | -.190 | **-.634***** | -.218 | **-.516***** | .227 | **.588***** | -.034 | **.576***** | .133 | **.602***** | .268 | **.379*** | .111 |
| Difficulties in emotion regulation | **-.383*** | .664*** | — | **-.376*** | **.587***** | **-.653***** | -.188 | **-.720***** | **-.559***** | **-.480**** | -.224 | **.616***** | -.167 | **.596***** | -.007 | **.646***** | .076 | **.517***** | .247 |
| Cognitive reappraisal | **.647***** | -.117 | -.272 | — | **-.316*** | **.480**** | .143 | **.374*** | .185 | **.412**** | **.435**** | -.305 | -.060 | **-.377*** | -.283 | **-.402*** | **-.424**** | -.259 | **-.362*** |
| Expressive suppression | **-.409*** | **.472**** | **.465**** | **-.449**** | — | **-.510***** | -.204 | **-.365*** | **-.441**** | **-.447**** | -.250 | .198 | -.223 | **.351*** | -.060 | .311 | .001 | .227 | .211 |
| **Secondary outcomes** | | | | | | | | | | | | | | | | | | | |
| Global self- esteem | **.530***** | **-.590***** | **-.604***** | .306 | **-.662***** | — | **.467**** | **.598***** | .310 | **.631***** | **.361*** | **-.516***** | -.095 | **-.543***** | .028 | **-.539***** | -.285 | **-.432**** | -.095 |
| Academic self- esteem | .220 | **-.335*** | -.272 | -.120 | -.052 | **.450**** | — | .195 | **.471**** | **.555***** | .174 | **-.338*** | .019 | **-.413**** | .054 | -.299 | -.160 | **-.534***** | -.159 |
| Social self- esteem | .120 | -.308 | **-.390*** | .090 | -.002 | .033 | .171 | — | .301 | **.565***** | .115 | **-.533***** | -.036 | **-.635***** | -.285 | **-.733***** | -.270 | **-.462**** | **-.358*** |
| Behavioural self-esteem | .297 | -.244 | -.255 | **.351*** | -.123 | .226 | **.322*** | -.059 | — | **.587***** | **.339*** | **-.527***** | .221 | **-.517***** | .090 | -.281 | .231 | **-.644***** | **-.328*** |
| Perseverance | **.559***** | **-.528***** | **-.575***** | **.380*** | **-.369*** | **.509**** | **.515***** | **.376*** | **.579***** | — | .310 | **-.482**** | -.022 | **-.629***** | -.165 | **-.455**** | -.129 | **-.648***** | **-.351*** |
| Assertiveness | **.543***** | -.271 | -.179 | **.596***** | **-.473**** | **.423**** | -.020 | -.009 | .284 | **.417**** | — | .046 | .078 | .004 | -.094 | .084 | .087 | -.149 | -.308 |
| Anxiety (child-report) | -.199 | **.552***** | **.595***** | .224 | .138 | **-.448**** | **-.511**** | **-.355*** | -.131 | **-.522***** | .193 | — | .269 | **.778***** | .049 | **.728***** | **.352*** | **.624***** | .143 |
| Anxiety (parent-report) | .099 | .077 | .178 | .094 | -.205 | .186 | -.143 | **-.382*** | -.162 | -.256 | .217 | **.487**** | — | .054 | **.387*** | .107 | **.550***** | .075 | .084 |
| Depression (child-report) | -.174 | **.499**** | **.628***** | .002 | **.358*** | **-.599***** | **-.429**** | -.313 | -.227 | **-.526***** | -.011 | **.749***** | .296 | — | .207 | **.773***** | .290 | **.622***** | **.367*** |
| Depression (parent-report) | .021 | .052 | .259 | .010 | -.084 | .028 | -.226 | **-.390*** | -.129 | -.269 | .169 | **.361*** | **.620***** | **.368*** | — | .231 | **.494**** | .041 | **.587***** |
| Internalising (child-report) | -.157 | **.586***** | **.512**** | .099 | .135 | **-.373*** | **-.418**** | **-.399*** | **-.346*** | **-.540***** | .124 | **.800***** | **.487**** | **.641***** | **.384*** | — | **.466**** | **.430**** | .231 |
| Internalising (parent-report) | .089 | .032 | .106 | -.056 | -.109 | .136 | .037 | -.267 | -.092 | -.203 | .057 | .220 | **.713***** | .166 | **.760***** | **.364*** | — | .062 | **.358*** |
| Externalising (child-report) | -.100 | **.381*** | **.435**** | .064 | -.010 | -.309 | **-.547***** | -.266 | **-.489**** | **-.554***** | .176 | **.534***** | .256 | **.619***** | **.329*** | **.602***** | .132 | — | **.440**** |
| Externalising (parent-report) | .053 | .110 | .291 | .001 | -.086 | .182 | .046 | -.086 | **-.349*** | -.274 | .164 | .235 | **.401*** | .212 | **.403*** | .212 | **.361*** | **.448**** | — |

*Note.* Correlations above the diagonal are for the control group (*n* = 40); correlations below the diagonal are for the intervention group (*n* = 38). *p* < .05 = *, *p* < .01 = **, *p* < .001 = ***; significant correlations are bolded for clarity.

*Supplementary Table 7.* Correlations between all outcome variables at follow-up (whole sample)

|  | **1** | **2** | **3** | **4** | **5** | **6** | **7** | **8** | **9** | **10** | **11** | **12** | **13** | **14** | **15** | **16** | **17** | **18** |
| --- | --- | --- | --- | --- | --- | --- | --- | --- | --- | --- | --- | --- | --- | --- | --- | --- | --- | --- |
| **Primary outcomes** | | | | | | | | | | | | | | | | | | |
| Productive coping | — |  |  |  |  |  |  |  |  |  |  |  |  |  |  |  |  |  |
| Non-productive coping | **-.346**** | — |  |  |  |  |  |  |  |  |  |  |  |  |  |  |  |  |
| Difficulties in emotion regulation | **-.385***** | **.667***** | — |  |  |  |  |  |  |  |  |  |  |  |  |  |  |  |
| Cognitive reappraisal | **.440***** | -.146 | -.216 | — |  |  |  |  |  |  |  |  |  |  |  |  |  |  |
| Expressive suppression | **-.224*** | **.408***** | **.481***** | **-.374***** | — |  |  |  |  |  |  |  |  |  |  |  |  |  |
| **Secondary outcomes** | | | | | | | | | | | | | | | | | | |
| Global self- esteem | **.564***** | **-.614***** | **-.594***** | **.247*** | **-.343**** | — |  |  |  |  |  |  |  |  |  |  |  |  |
| Academic self- esteem | **.355**** | **-.406***** | **-.456***** | .061 | -.099 | **.627***** | — |  |  |  |  |  |  |  |  |  |  |  |
| Social self- esteem | **.227*** | **-.531***** | **-.559***** | .210 | **-.412***** | **.449***** | **.355**** | — |  |  |  |  |  |  |  |  |  |  |
| Behavioural self-esteem | **.385***** | **-.403***** | **-.560***** | .137 | **-.269*** | **.345**** | **.281*** | .089 | — |  |  |  |  |  |  |  |  |  |
| Perseverance | **.530***** | **-.487***** | **-.635***** | **.305**** | **-.490***** | **.575***** | **.419***** | **.523***** | **.369***** | — |  |  |  |  |  |  |  |  |
| Assertiveness | **.555***** | **-.237*** | **-.278*** | **.429***** | **-.265*** | **.417***** | .113 | **.231*** | **.278*** | **.453***** | — |  |  |  |  |  |  |  |
| Anxiety (child-report) | -.149 | **.552***** | **.647***** | .062 | **.281*** | **-.493***** | **-.512***** | **-.448***** | **-.247*** | **-.329**** | .095 | — |  |  |  |  |  |  |
| Anxiety (parent-report) | -.055 | **.314**** | .148 | .133 | .049 | -.182 | -.107 | -.266* | .021 | -.193 | .012 | **.314**** | — |  |  |  |  |  |
| Depression (child-report) | **-.278*** | **.580***** | **.625***** | -.222 | **.315**** | **-.468***** | **-.445***** | **-.521***** | **-.425***** | **-.523***** | -.030 | **.671***** | **.230*** | — |  |  |  |  |
| Depression (parent-report) | -.162 | **.258*** | .196 | -.080 | **.229*** | -.168 | -.123 | **-.257*** | -.164 | **-.326**** | .024 | .150 | **.618***** | **.395***** | — |  |  |  |
| Internalising (child-report) | **-.303**** | **.623***** | **.654***** | -.119 | **.270*** | **-.586***** | **-.581***** | **-.539***** | **-.263*** | **-.578***** | -.108 | **.764***** | **.333**** | **.662***** | **.244*** | — |  |  |
| Internalising (parent-report) | .001 | .222 | .202 | -.153 | .177 | -.169 | -.080 | **-.392***** | .153 | **-.254*** | -.046 | **.260*** | **.606***** | **.312**** | **.603***** | **.415***** | — |  |
| Externalising (child-report) | **-.268*** | **.453***** | **.612***** | -.037 | **.244*** | **-.416***** | **-.516***** | **-.314**** | **-.596***** | **-.557***** | -.053 | **.561***** | .197 | **.606***** | **.303**** | **.611***** | .131 | — |
| Externalising (parent-report) | -.181 | **.267*** | **.289*** | -.148 | .098 | -.133 | -.083 | **-.226*** | **-.406***** | -.196 | -.106 | .157 | **.297**** | **.326**** | **.468***** | .192 | **.336**** | **.427***** |

*Note. N* = 78. *p* < .05 = *, *p* < .01 = **, *p* < .001 = ***; significant correlations are bolded for clarity.

*Supplementary Table 8.* Correlations between all outcome variables at follow-up by condition

|  | **1** | **2** | **3** | **4** | **5** | **6** | **7** | **8** | **9** | **10** | **11** | **12** | **13** | **14** | **15** | **16** | **17** | **18** | **19** |
| --- | --- | --- | --- | --- | --- | --- | --- | --- | --- | --- | --- | --- | --- | --- | --- | --- | --- | --- | --- |
| **Primary outcomes** | | | | | | | | | | | | | | | | | | | |
| Productive coping | — | -.225 | **-.320*** | .261 | -.054 | **.417**** | **.317*** | .188 | **.368*** | **.492**** | **.627***** | -.074 | .128 | -.202 | -.111 | -.253 | .171 | **-.347*** | -.131 |
| Non-productive coping | **-.470**** | — | **.687***** | -.050 | **.521***** | **-.620***** | **-.450**** | **-.633***** | **-.484**** | **-.645***** | -.193 | **.623***** | **.336*** | **.667***** | .**314*** | **.733***** | .354* | **.604***** | **.328*** |
| Difficulties in emotion regulation | **-.457**** | **.667***** | — | -.133 | **.482**** | **-.719***** | **-.622***** | **-.693***** | **-.709***** | **-.663***** | **-.439**** | **.641***** | .053 | **.646***** | .127 | **.714***** | .220 | **.717***** | **.415**** |
| Cognitive reappraisal | **.575***** | -.227 | -.304 | — | -.198 | .153 | .082 | **.330*** | -.092 | .236 | .137 | .058 | .250 | -.303 | -.133 | -.196 | -.251 | -.079 | -.078 |
| Expressive suppression | **-.329*** | **.358*** | **.510**** | **-.469**** | — | **-.332*** | -.180 | -.532*** | -.211 | **-.356*** | -.071 | **.409**** | -.029 | **.325*** | -.014 | **.385*** | .259 | .257 | .105 |
| **Secondary outcomes** | | | | | | | | | | | | | | | | | | | |
| Global self- esteem | **.696***** | **-.610***** | **-.464**** | **.320*** | **-.361*** | — | **.768***** | **.537***** | **.489**** | **.594***** | .359* | **-.664***** | -.195 | **-.558***** | -.203 | **-.657***** | -.306 | **-.609***** | -.229 |
| Academic self- esteem | **.410*** | **-.353*** | -.286 | .044 | -.064 | **.495**** | — | **.452**** | **.476**** | **.511***** | .298 | **-.517***** | .046 | **-.478**** | -.077 | **-.599***** | -.095 | **-.607***** | -.097 |
| Social self- esteem | .257 | **-.440**** | **-.390*** | .120 | **-.341*** | **.366*** | .281 | — | .269 | **.610***** | .184 | **-.559***** | -.106 | **-.692***** | -.145 | **-.619***** | -.379* | **-.466**** | **-.384*** |
| Behavioural self-esteem | **.420**** | -.302 | **-.368*** | **.362*** | **-.349*** | .184 | .026 | -.118 | — | **.509***** | .368* | **-.485**** | .062 | **-.523***** | -.079 | **-.576***** | .144 | **-.784***** | **-.528***** |
| Perseverance | **.565***** | **-.378*** | **-.606***** | **.370*** | **-.577***** | **.578***** | **.398*** | **.430**** | .262 | — | .437** | **-.313*** | -.102 | **-.548***** | -.346* | **-.587***** | -.180 | **-.692***** | **-.359*** |
| Assertiveness | **.517***** | -.282 | -.159 | **.590***** | **-.349*** | **.465**** | -.007 | .265 | .230 | **.471**** | — | -.038 | .171 | -.082 | .015 | -.209 | .145 | -.281 | -.089 |
| Anxiety (child-report) | -.220 | **.484**** | **.651***** | .067 | .197 | -.319 | **-.535***** | -.315 | .054 | **-.333*** | .207 | — | .235 | **.672***** | -.010 | **.763***** | .222 | **.609***** | .155 |
| Anxiety (parent-report) | -.266 | .290 | .288 | .026 | .117 | -.169 | -.307 | **-.474**** | -.040 | -.310 | -.121 | **.423**** | — | .245 | **.610***** | .176 | **.630***** | .122 | .071 |
| Depression (child-report) | **-.385*** | **.470**** | **.620***** | -.153 | **.350*** | **-.370*** | **-.397*** | **-.328*** | -.271 | **-.561***** | .008 | **.691***** | .207 | — | **.388*** | **.750***** | **.490**** | **.643***** | **.478**** |
| Depression (parent-report) | -.212 | .198 | .281 | -.039 | **.400*** | -.135 | -.172 | **-.376*** | -.269 | **-.322*** | .031 | **.329*** | **.634***** | **.412*** | — | .181 | **.624***** | .223 | **.402*** |
| Internalising (child-report) | **-.350*** | **.516***** | **.569***** | -.057 | .194 | **-.518***** | **-.597***** | **-.439**** | .137 | **-.563***** | -.029 | **.762***** | **.552***** | **.565***** | .319 | — | **.328*** | **.722***** | .235 |
| Internalising (parent-report) | -.107 | .142 | .170 | -.098 | .123 | -.073 | -.104 | **-.393*** | .155 | -.268 | -.137 | .284 | **.631***** | .186 | **.610***** | **.487**** | — | .093 | .238 |
| Externalising (child-report) | -.179 | .287 | **.457**** | -.001 | .243 | -.207 | **-.442**** | -.119 | **-.353*** | **-.405*** | .135 | **.494**** | .309 | **.576***** | **.402*** | **.461**** | .145 | — | **.474**** |
| Externalising (parent-report) | -.232 | .205 | .173 | -.200 | .104 | -.049 | -.057 | -.093 | -.272 | -.087 | -.124 | .172 | **.565***** | .150 | **.533***** | .165 | **.435**** | **.401*** | — |

*Note.* Correlations above the diagonal are for the control group (*n* = 40); correlations below the diagonal are for the intervention group (*n* = 38). *p* < .05 = *, *p* < .01 = **, *p* < .001 = ***; significant correlations are bolded for clarity.
